# Adaptation of the pathogen, *Pseudomonas syringae*, during experimental evolution on a native vs. alternative host plant

**DOI:** 10.1111/mec.14060

**Published:** 2017-03-13

**Authors:** Sean Meaden, Britt Koskella

**Affiliations:** ^1^ University of Exeter Penryn Campus Penryn Cornwall TR11 4EH UK; ^2^ Department of Integrative Biology University of California, Berkeley Berkeley CA 94720 USA

**Keywords:** Arabidopsis, bacteriophage, experimental evolution, host specialization, phyllosphere, plant pathogen, reservoir host, tomato

## Abstract

The specialization and distribution of pathogens among species has substantial impact on disease spread, especially when reservoir hosts can maintain high pathogen densities or select for increased pathogen virulence. Theory predicts that optimal within‐host growth rate will vary among host genotypes/species and therefore that pathogens infecting multiple hosts should experience different selection pressures depending on the host environment in which they are found. This should be true for pathogens with broad host ranges, but also those experiencing opportunistic infections on novel hosts or that spill over among host populations. There is very little empirical data, however, regarding how adaptation to one host might directly influence infectivity and growth on another. We took an experimental evolution approach to examine short‐term adaptation of the plant pathogen, *Pseudomonas syringae* pathovar *tomato*, to its native tomato host compared with an alternative host, *Arabidopsis*, in either the presence or absence of bacteriophages. After four serial passages (20 days of selection *in planta*), we measured bacterial growth of selected lines in leaves of either the focal or alternative host. We found that passage through *Arabidopsis* led to greater within‐host bacterial densities in both hosts than did passage through tomato. Whole genome resequencing of evolved isolates identified numerous single nucleotide polymorphisms based on our novel draft assembly for strain PT23. However, there was no clear pattern of clustering among plant selection lines at the genetic level despite the phenotypic differences observed. Together, the results emphasize that previous host associations can influence the within‐host growth rate of pathogens.

## Introduction

Many pathogenic organisms are generalists, capable of infecting multiple host species (Woolhouse 2001). However, how selection across multiple hosts influences the evolution of pathogen growth and optimal virulence remains unclear, and has rarely been examined empirically. In particular, there is a predicted trade‐off between generalism and specialism when the ability to infect an alternative host leads to reduced replication efficiency in the original host (Benmayor *et al*. 2009). Established theory demonstrates that parasites evolve to an optimum level of virulence that maximizes parasite transmission (Van Baalen & Sabelis 1995; Anderson & May 2009), and this idea has been well supported by empirical results (Thrall & Burdon 2003; Jensen *et al*. 2006; de Roode *et al*. 2008; Bérénos *et al*. 2009; Müller *et al*. 2009). Given that these optima are likely to vary among host genotypes and species, a pathogen infecting a new or alternative host could be expected to show suboptimal fitness. However, many studies, particularly those carried out on viruses, demonstrate that generalists can have at least as high fitness as specialists in the same host (Elena *et al*. 2009; Remold 2012). Moreover, multihost pathogens are extremely common and their evolution is expected under a theoretical framework when transmission between hosts is high (Woolhouse 2001; Gandon 2004). In this study, we sought to understand the effects that short‐term passaging and potential adaptation to one host has on growth of a pathogen in an alternative host.

Understanding evolution across hosts is important, as the maintenance of pathogens on wild plants can act as a reservoir of infection for many important crop diseases, including bacterial, viral and fungal pathogens (Mueller *et al*. 2012; Malcolm *et al*. 2013; Thinakaran *et al*. 2015). In this case, selection within reservoir hosts could lead to the attenuation of virulence on agricultural hosts (if a trade‐off between growth in the two hosts exists), or increased virulence on agricultural hosts (if there exist high transmission rates in the reservoir that offset any costs of increased exploitation of the agricultural host). Additionally, for bacterial and fungal pathogens, there are likely other biotic factors influencing adaptation to hosts, including infection by viral parasites (also known as hyperparasites). Lytic bacteriophage viruses (phages) are known to exert considerable selective pressures on bacterial populations, with potential evolutionary trade‐offs in terms of within‐host bacterial growth and virulence, typically via the modification of bacterial surface receptors used for phage binding (Filippov *et al*. 2011; Hall *et al*. 2012; Koskella & Taylor 2015; Meaden *et al*. 2015). Given the potential impacts of host heterogeneity and within‐host selection, our ability to predict pathogen adaptation requires better incorporation of these ecological complexities.

One powerful way to test predictions about pathogen adaptation under varying biotic environments is through experimental evolution (Ebert 1998; Elena 2016). For example, after four generations of experimental passaging, the foliar necrotroph, *Stemphylium solani*, showed increased rates of infection but no change in virulence across 12 clover lineages (Gilbert & Parker 2010). Experimental evolution of the tobacco etch potyvirus (TEV) under either constant or alternating host environments demonstrated that evolutionary history of the pathogen influences virulence, but found no cost to generalism (Bedhomme *et al*. 2012). The bacterial wilt pathogen, *Ralstonia solanacearum*, increased its fitness on both tomato and bean hosts, but increased most on the foreign host to which the pathogen was not well‐adapted initially (Guidot *et al*. 2014). Coupled with resequencing experiments, this approach can also determine the molecular underpinnings of adaptation (Bartoli *et al*. 2016). By starting with a single ancestral pathogen clone, it is possible to remove the confounding effects of differences in genomic architecture that may influence the evolution of certain traits. Furthermore, it is possible to identify parallel mutations (i.e. mutations occurring within the same gene or pathway) across replicate populations, the presence of which would be highly suggestive of adaptive mutations rather than an artefact of genetic drift (Elena & Lenski 2003). This phenomenon has been demonstrated in a number of plant pathogen systems (e.g. Pitman *et al*. 2005; Trivedi & Wang 2014).

In this study, we examined pathogen adaptation to a distant host, *Arabidopsis*, relative to adaptation to its native host species, tomato, after short‐term passaging. We took an experimental evolution approach using a single starting clone of the bacterium *Pseudomonas syringae* pv. tomato PT23 (*Pst*) by serially passaging the pathogen on each host, in either the presence or absence of phages. After four experimental passages, equating to 20 days of selection *in planta*, we assayed the derived lineages on both the plant species used for the passage and the alternative host, allowing the comparison of bacterial growth on both hosts. Whole genome resequencing was performed to identify genes or pathways showing parallel evolution across replicates and to provide a novel, draft assembly of the plant pathogen *P. syringae* pv. tomato PT23. We predicted that bacteria experimentally evolved on one host would show reduced fitness when assayed on the alternative host, and that this effect may be more pronounced for the non‐native host lines. We also predicted that the presence of phages would reduce virulence and/or constrain adaptation to the plant host based on reductions in bacterial population sizes, and the fitness costs associated with resistance mechanisms.

## Materials and methods

### Study system

*Pseudomonas syringae* represents an important plant pathogen species complex, collectively infecting numerous plant species and acting as a major agricultural pest (Mansfield *et al*. 2012). It also represents a true generalist, as it is frequently isolated not just from plant infections but diverse environmental sources (Morris *et al*. 2008). Moreover, these nonagricultural reservoirs likely provide a source population for the evolution of novel plant pathovars (Monteil *et al*. 2013). *Pseudomonas syringae* pv. tomato (Pst) has been extensively studied as a model plant pathogen for elucidating the molecular basis of infection, as it infects both its natural tomato host, *Solanum lycopersicum*, and the model organism *Arabidopsis thaliana*, resulting in necrotic lesions surrounded by chlorotic tissue (Mittal & Davis 1995). A number of studies have also used *P. syringae* to test hypotheses in evolutionary biology both in the laboratory (Lythgoe & Chao 2003; Koskella *et al*. 2012) and in the field (Kniskern *et al*. 2011; Koskella *et al*. 2011; Nowell *et al*. 2016). Finally, a completed, gold standard genome of the Pst pathovar DC3000 is available, providing a useful resource for genomic analyses (Buell *et al*. 2003). The closely related pathovar PT23 was used in this study due to preliminary data suggesting that this strain supports higher phage populations than DC3000 (data not shown) and existing literature identifying virulence factors (Preston 2000) and their potential role in Money Maker infections (Badel *et al*. 2006).

### Passage experiment

A single colony of Pst PT23 was picked and cultured overnight in King's B broth (KB) to serve as the ancestral strain. Eighteen tomato (*Solanum lycopersicum* cultivar Moneymaker) and 18 *Arabidopsis thaliana* (ecotype Columbia) plants were grown for 5 weeks in a controlled temperature (CT) room with 80% relative humidity, 24 °C and 15‐h photoperiod. Bacterial inocula were prepared using 25 mL of overnight ancestral Pst PT23 culture grown in KB broth. The culture was centrifuged at 2800 *g* for 6 min and resuspended in 20 mL MgCl_2_ buffer, with this process being repeated twice as a ‘washing’ step to remove residual media, leaving a concentration of approximately 8 × 10^7^ CFU/mL.

Treatments of bacteria only, bacteria and phage co‐inoculum, or buffer only were assigned randomly to each of 18 plants per species (six replicates per treatment). Each 1 mL suspension consisted of 500 μL of bacteria and either 500 μL of buffer or 500 μL of phage, with control plants receiving buffer only. The phage solution was a clonal suspension of a Pst PT23 plaque‐forming, lytic phage (FRS, described in Meaden *et al*. 2015) at a concentration of approximately 5 × 10^6^ PFU/mL. Solutions were mixed immediately prior to inoculation into the abaxial side of the leaf using a blunt end syringe (Wei *et al*. 2007). Concurrently, in vitro control lines (one line matched to each plant line) were inoculated into 8 mL agar slants (KB broth supplemented with 0.6% agar) and placed in the CT room among the experimental plants. After 5 days, a 1 cm hole punch was used to collect leaf samples from inoculated leaves of each plant. Samples were dipped in 0.1 m surface sterilization buffer (0.02% Tween 20, 1% Sodium hypochlorite) for 5 s to remove epiphytic bacteria, dipped in sterile ddH_2_O for 5 s and stored at −20 °C in 1 mL of phosphate buffer (pH 7, supplemented with peptone and glycerol). Similarly, for the agar slants, a stab was taken with a 1000‐μL pipette from each slant and added to 1 mL of phosphate buffer and stored at −20 °C.

To determine bacterial densities, samples were snap‐thawed at 37 °C and homogenized in a Fast‐Prep tissue lyser (MP Biomedicals) with two ceramic beads. In vitro samples underwent a similar process with the tissue lysis step being replaced with vortexing for 5 s. Following plating on KB agar (supplemented with 25 μg/mL the antifungal nystatin), 100 colonies were picked at random to exclude phages and plant hormones and then combined and suspended in 1 mL of 10 mm MgCl_2_. This suspension formed the inoculation for the next cohort of plants, which had been planted approximately 1 week later than the previous cohort. By combining bacterial colonies grown on KB plates, rather than bacteria collected directly from leaves, we were able to avoid passaging any other bacteria that may be growing in/on leaves, as well as plant hormones that might alter plant defences. This transfer process was conducted four times, including phage co‐inoculation, leaving the bacterial populations in the plant or the matched in vitro environments, for a total of 20 days.

### Assay experiments

For the phenotypic assays and sequencing, a single colony was picked from each line after the 4th transfer (day 20), cultured overnight and frozen in 20% glycerol for future use. For seven lines, samples from the third transfer were used due to overgrowth of other, unknown bacterial colonies or the absence of any colonies at the final time point. Each line was cultured from frozen for 48 h and then prepared using the same methods as described for the passage experiment. Each suspension was standardized to an optical density (600 nm) of ~0.05, using a spectrophotometer (PowerWave XS, Biotek, USA; ~6 × 10^7^ CFU/mL based on a previous standard curve), and inoculated into a new cohort of plants in the same manner as before, this time excluding phage co‐inoculation. The design of the experiment allowed each line to be inoculated into both a tomato and *Arabidopsis* plant, such that each strain was assayed on both a host plant from the species they were passaged on and a plant from the alternative host species, with a total of 72 plants including the lines adapted to media (i.e. 12 lines from *Arabidopsis*, 12 lines from Tomato and 12 from media inoculated into one of each host). Leaf samples were collected after 24, 72 and 192 h per plant from independent leaves. Note that the assay time was allowed to run for a longer sampling period than the original transfers.

Bacterial plating was conducted first using a drop‐plate method (Chen *et al*. 2003) to determine appropriate plating dilution, and then by plating 50 μL of each sample at the appropriate dilution for colony counting at a better resolution. For growth curve analyses, each line was cultured from frozen stocks in KB media for 48 h to reach stationary phase. Each culture was diluted 50 times into ddH_2_O, and 20 μL was added to 180 μL of KB broth in a 96‐well plate. Optical density (600 nm) readings were recorded for 48 h, and these growth curves were used to infer fitness parameters such as maximum growth rate and final density. To screen for the evolution of phage resistance, 12 colonies were picked from the fourth transfer and cultured in a 96‐well plate with 100 μL KB broth. 20 μL of ancestral phage stock was streaked vertically across an agar plate, with bacterial cultures streaked horizontally across the plate using a pin replicator. This assay allows a binary score of phage infectivity or resistance as shown by either a continuous line of growth across the phage streak or a band of inhibited growth. Plates were scored after incubation at 28 C for 24 h.

### Statistical analysis

We first compared bacterial population sizes (log 10‐transformed) of all experimental lines, including the in vitro lines, in each of the two host species after 24, 72 and 192 h using a repeated measures general linear model (note that all results were qualitatively the same when a general linear model was used with plant ID built in as a random factor). We then reran the analysis while excluding the 192‐h time point, given the extremely low bacterial densities observed across all treatments at this point, and then again while excluding the in vitro treatment to specifically test the effect of selection *in planta*. To compare the in vitro growth rate of the evolved lines, general linear models were performed using densities in vitro as the dependent variable, and experimental treatment (phage/no phage and tomato/*Arabidopsis*) as fixed terms. *In planta* assay analyses comparing bacterial growth were run in SPSS version 24, and the in vitro assay and SNP filtering analyses were run in R version 3.2.4.

### 16S sequencing

To confirm our accuracy in identifying Pst based on colony morphology on hard agar, we picked 117 colonies (12 per plate from a subset of 10 plant samples, three of which failed sequencing) and performed PCR of the 16S rRNA gene with the primers 27f and 907r following the protocol of Frank *et al*. (2008). Sequencing was performed at Source Biosciences (Oxford, UK). A number of the plated samples from the passage and assay parts of the experiment contained colonies with different morphology to *P. syringae*, which we suspected to be other epiphytic bacterial species. To identify them, the same PCR protocol was used as above and sample clean‐up was performed with Exo‐Sap (Affymetrix, USA). Forward Sanger sequencing was performed on 10 samples at the Sheffield University Core Genomic Centre (Sheffield, UK). For both batches, reads were blasted against the ncbi database.

### Whole genome resequencing

DNA extractions were performed using 48‐h cultures from the frozen clones selected at the end of the passaging experiment. 200 μL was pelleted by centrifugation, resuspended in 200 μL of nuclease free water and DNA extracted using a DNeasy kit (Qiagen). Clean‐up and concentration steps were performed using AMPure beads and genomic DNA clean and concentrator kit (Zymo Research, USA) and quantified using a Qubit fluorimeter. 100‐bp paired‐end Illumina sequencing was conducted on a HiSeq 2500 at the University of Exeter Sequencing Centre. Library preparation was performed following the nextera xt version 2 protocol. Sequences were trimmed using sickle (default parameters; Joshi & Fass 2011), and sequences from the ancestral samples were assembled with spades 3.9. (Bankevich *et al*. 2012). Plasmids were independently assembly using the ‘plasmid’ option with spades 3.9. Whole genome and plasmid assemblies were annotated in rast (Aziz *et al*. 2008) and the remaining samples mapped using bwa (Li & Durbin 2009). Variant calling was performed on the resulting alignments using the gatk haplotypecaller (McKenna *et al*. 2010). Tablet (Milne *et al*. 2010) was used for manual inspection of variants and read alignments with an error rate of 10% false positives following manual inspection of a subsample of SNP calls (*n* = 20). Reads from the ancestral sample were mapped to the reference assembly and all SNPs at those positions were discarded as false positives. The remaining SNPs were filtered on a phred score of over 99 and read coverage of between 15 and 100 reads and then linked to the rast annotation. To identify SNPs associated with plant adaptation, we took two filtering approaches. First, we filtered all SNPs present in the media adapted samples, based on position, and used this data set for hierarchical clustering and SNP counts within samples. We then took a more conservative approach and filtered by gene annotation, therefore removing all SNPs that occurred in genes of similar function in both media and plant samples.

As this was the first time the PT23 genome had been fully sequenced, we have deposited the draft genome in the ncbi database (BioProject ID PRJNA357646). Comparative genomics between PT23, *P. syringae* pv. tomato DC3000, *P. syringae* pv. syringae B728 and *P. syringae* pv. actinidae was performed using brig 0.95 (Alikhan *et al*. 2011) and blast+ 2.4 (Camacho *et al*. 2009). Whole genome alignment between PT23 and *P. syringae* pv. tomato DC3000 was performed using the progressivemauve algorithm and contigs re‐ordered in mauve (Darling *et al*. 2010).

## Results

Our experiment aimed to assess the impact of short‐term adaptation on an alternative host to growth on a native host, and vice versa. After passaging a single clone of PT23 through either tomato (the native host) or *Arabidopsis* (an alternative host), in the presence or absence of phage co‐inoculation, we assayed pathogen growth on both its passage host and nonpassage host in the absence of phage co‐inoculation. We then used whole genome resequencing scans to associate mutations with any derived phenotypes.

### Effect of selection environment on growth in plants

**Figure 1:**
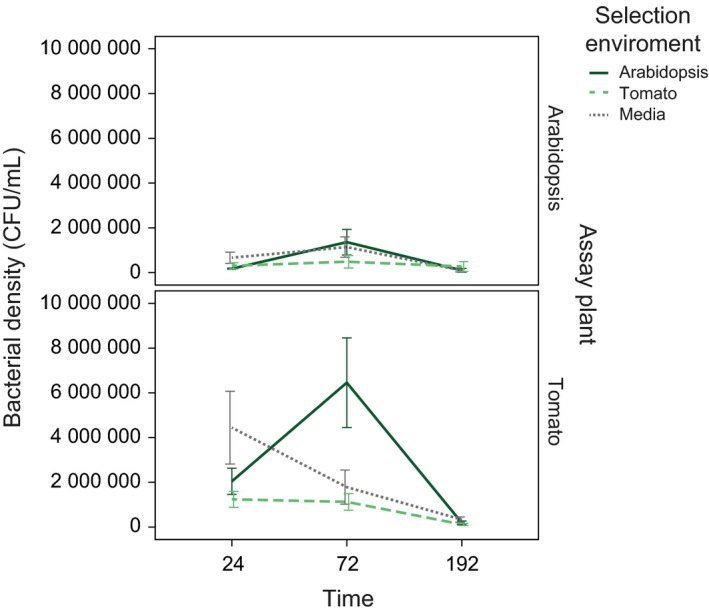
Results from the final assay, in which all experimental evolution lines were grown on either Arabidopsis (top panel) or tomato (bottom panel), and mean bacterial densities (colony‐forming units) were compared across each of three time points. 72 plants were used in total with one treatment line per plant. Bacterial lines evolved in Arabidopsis are shown in solid dark green, those evolved in tomato in long‐dashed light green, and those evolved in media in short‐dashed grey. Error bars represent ±1 standard error of the mean. [Colour figure can be viewed at http://wileyonlinelibrary.com]

After four passages (20 days) in either *Arabidopsis*, Tomato, or KB media controls, we observed an effect of selection environment on bacterial density when isolates were grown *in planta*. When bacterial densities (log10‐transformed) were compared across all three time points (24, 72, and 192 h) using a repeated measures general linear model, we found a clear main effect of assay plant on bacterial density observed (*F*_1,44_ = 18.958, *P *< 0.0001) as well as a strong effect of sampling time (*F*_1,44_ = 61.966, *P *< 0.0001), but no effect of selection environment (*F*_2,44_ = 1.713, *P *= 0.192) or phage treatment (*F*_1,44_ = 0.431, *P *= 0.515), and no significant interaction effects. Specifically, bacteria were able to reach higher densities when grown in Tomato than *Arabidopsis*, regardless of which environment that isolate had been selected in (no interaction between assay plant and time was observed, *F*_1,44_ = 2.086, *P *= 0.156; Fig. 1). This result was consistent with the density results found over the course of passaging (Fig. S2, Supporting information).

Given the strong effect of time, as bacterial densities dropped significantly between 72 and 192 h, we reran the analysis after removal of the final time point (in which most densities had decreased towards 0). This allowed us to determine the influence of selection environment on initial growth within the plant prior to lesion formation and leaf drying. In this case, we found no significant effect of time on log10‐transformed densities (*F*_1,53_ = 0.077, *P *= 0.783), but significant effects of both assay plant (*F*_1,53_ = 22.067, *P *< 0.0001) and selection environment (*F*_2,53_ = 4.298, *P *= 0.019). Again, we found no effect of phage treatment (*F*_1,53_ = 0.367, *P *= 0.547), and, importantly, no interaction between selection environment and assay plant (*F*_2,53_ = 0.640, *P *= 0.531), indicating that selection in the *Arabidopsis* host plant lead to a more rapidly growing bacterial phenotype in either assay plant. This result remained qualitatively the same after removal of the media selection environment, indicating the largest differences were observed between selection in the two plant host environments, and was also qualitatively similar after removal of phage treatment from the model.

Despite the differences in density observed *in planta*, we found no differences among the lines in maximum growth rate (GLM, *F*_1,30_ = 0.59, *P* = 0.56) or final density after 48 h (*F*_1,30_ = 1.36, *P* = 0.272) of incubation in vitro. Here, bacterial lines from Tomato and *Arabidopsis* were cultured in nutrient media and analysed using an optical density plate reader (OD600) that monitored growth over the course of 24 h. Furthermore, when we tested for nongenetic effects of plant environment on growth by repeating the assays in tomato with bacteria collected from a subsample of either the *Arabidopsis* (*N* = 5) or tomato (*N* = 5) lines after only the first transfer, we found no difference in bacterial densities between tomato and *Arabidopsis* after 48 h growth (GLM, *F*_1,9_ = 0.414, *P* = 0.538).

### Effect of selection environment on molecular evolution

**Figure 2:**
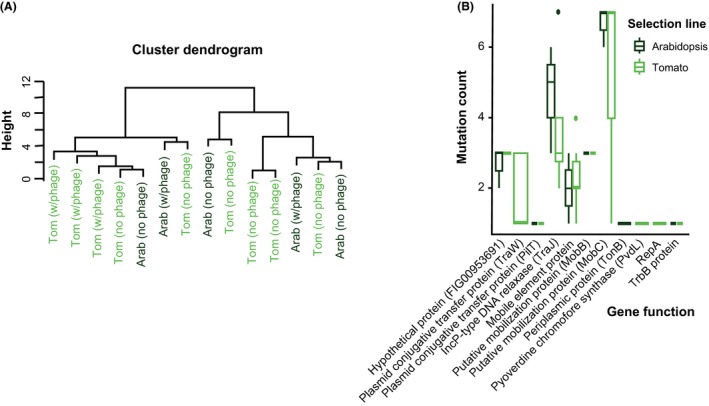
Comparison of observed mutations across the Arabidopsis (dark green) and Tomato (light green) selection lines based on location. (A) Hierarchical clustering of samples based on single nucleotide polymorphisms (SNPs) at the gene level, and (B) comparison of putative mutations across Arabidopsis and tomato lines that were not observed in the media selection lines. [Colour figure can be viewed at http://wileyonlinelibrary.com]

To build on these phenotypic results, we performed genome scans using a resequencing approach to link the observed changes in growth, depending on selection environment, to observations at the molecular level. While we did identify a number of SNPs that seemed to be associated with adaptation *in planta* relative to in vitro growth, hierarchical clustering of SNPs across plant lines at the genetic level did not group any samples consistently together (Fig. 2A). Despite some of these mutations being related to plasmid transfer and conjugation, mapping of reads to an assembled plasmid and manual inspection found no candidate SNPs within the plasmid itself. Our second SNP analysis, that filtered samples based on predicted gene functionality rather than genomic position, identified four SNPs with annotations that occurred only in the plant adapted selection lines. These were a hydrogen peroxide‐inducible genes activator (HPIGA herein) and periplasmic protein (TonB) in two *Arabidodopsis* lines and a pyoverdine precursor (PvdL) and an alcohol dehydrogenase (EC 1.1.1.28) in two tomato lines. Although these mutations did occur in parallel lines, two of the genes are associated with siderophore production and transport (PvdL and TonB) – a bacterial signalling system, while the other two regulate surface proteins (HPIGA) and metabolism (EC1.1.1.28), giving clues about in vivo microbial selection pressures but not providing clear evidence for parallel evolution. Taken together, our results suggest that a small number of de novo mutations may have been selected for and be responsible for the observed phenotypes, but that these mutations are not consistent within experimental treatments, so no clear genotype‐phenotype link can be drawn.

**Figure 3:**
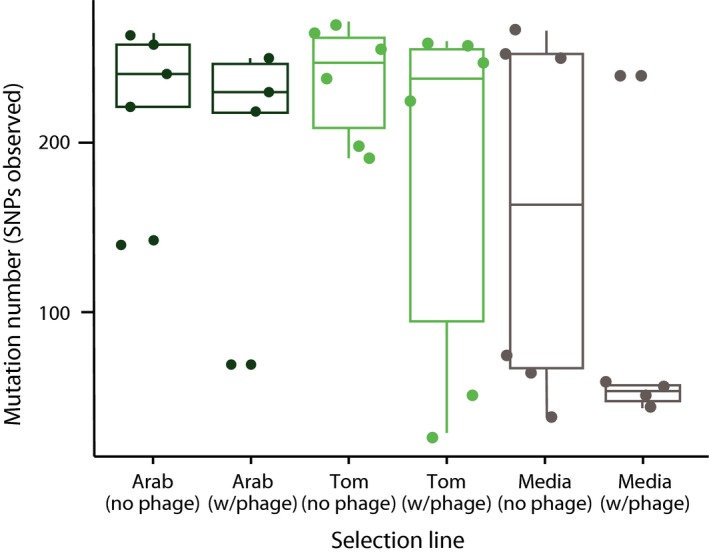
Absolute number of SNPs (independent of location) in the Arabidopsis, tomato and media environments, and depending on whether the selection line included phage‐mediated selection or not. [Colour figure can be viewed at http://wileyonlinelibrary.com]

In terms of the absolute number of mutations observed across selection environments, we found significantly more SNPs in lines evolved in plants compared to those evolved in media (Fig. 3; General linear model, *F*_1,32_ = 8.31, *P *< 0.01). However, no difference was observed when only the two plant environments were included in the analysis (*F*_1,20_ = 0.079, *P *= 0.78). In combination with the previous clustering results, this suggests that the phenotypic difference observed is not clearly related to change at the genetic level. Furthermore, in terms of the impact of phage selection on the molecular evolution of the pathogen, we observed slightly fewer SNPs in lines under phage‐mediated selection than those under phage relaxed selection (Fig. 3); however, this was only nearing significance (Main effect of phage, *F*_1,32_ = 3.32, *P* = 0.08). We found no interaction between phage treatment and selection environment on the number of SNPs observed (*F*_1,30_ = 1.25, *P* = 0.27).

### PT23 genome assembly and comparative genomics

**Figure 4:**
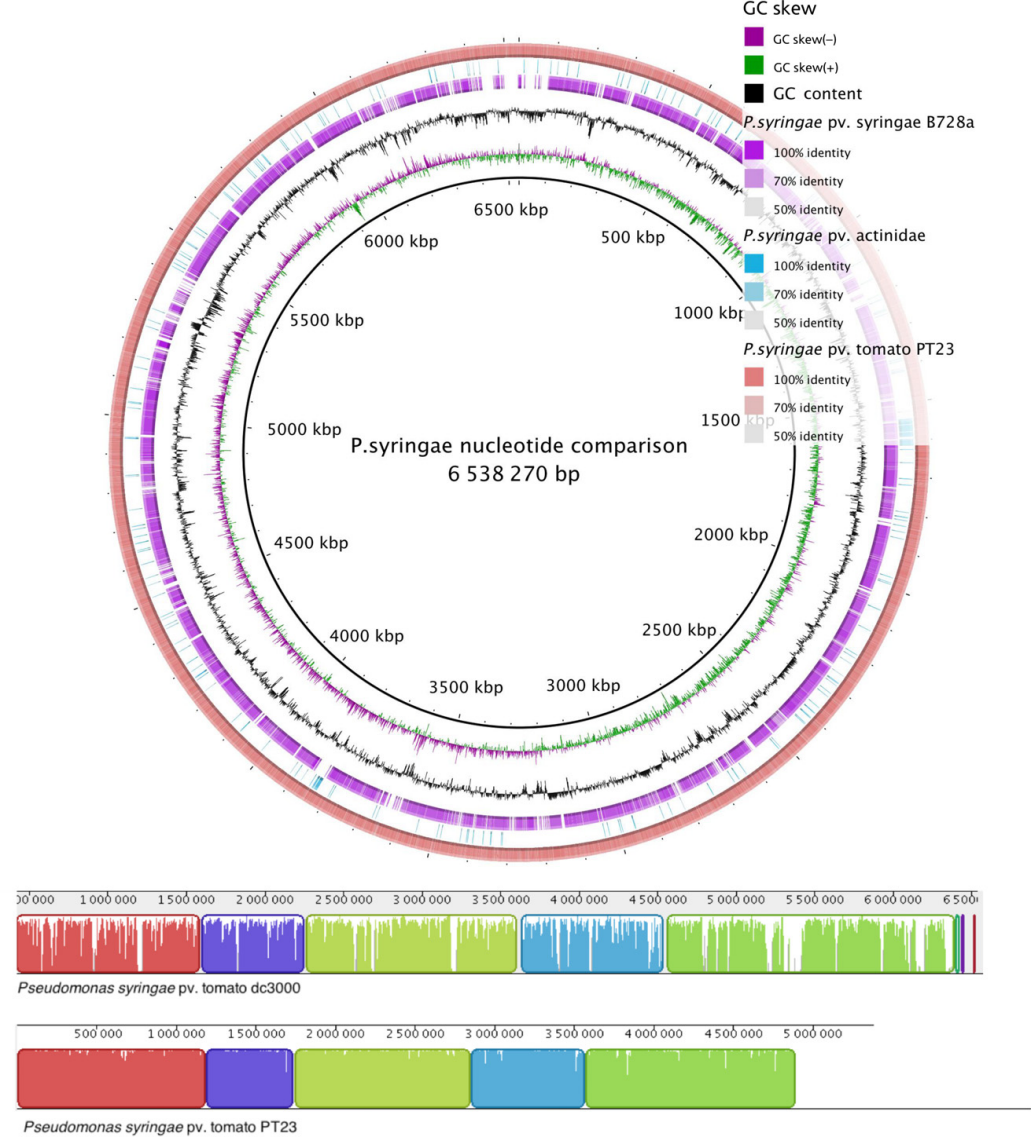
Whole genome alignments of PT23 and three well‐studied *P. syringae* pathovars. The completed assembly of *P. syringae* pv. tomato DC3000 was used as the reference genome (inner, black circle). PT23 (red, outer circle) shows high sequence homology with DC3000. Conversely, *P. syringae* pv. syringae (Purple, outer third circle) shows less homology and *P. syringae* pv. actinidae (Blue, outer second circle) shows very little homology, consistent with the differences in each pathovar's host phylogeny. A whole genome alignment of PT23 and DC3000 identified contiguous, co‐linear blocks of each genome (bottom panel alignment, coloured blocks) with varying sequence similarity (bar height inside each block). [Colour figure can be viewed at http://wileyonlinelibrary.com]

An additional aim of this experiment was to annotate our draft assembly, as there are no currently available PT23 genome assemblies available. Our PT23 assembly resulted in a 6.29 Mbp genome from 223 contigs with an N50 of 142 kb. The PT23 genome has a GC content of 58% and 5667 coding sequences. Our assembly showed very high sequence similarity with the closely related *P. syringae* pv. tomato DC3000 compared to *P. syringae* pv. syringae B728 and *P. syringae* pv. actinidae, which both infect different host species (Fig. 4). Annotation uncovered 113 genes putatively associated with virulence and disease, specifically resistance to many metal compounds including copper, arsenic, fluoroquinolone and bacteriocin production. Independent plasmid assembly and annotation suggests many of these putative copper‐resistance genes are located on a plasmid carrying a toxin–antitoxin system.

## Discussion

Understanding how within‐host evolution alters both fitness and molecular evolution is particularly important for pathogens with broad host range or pathogens that alternate among primary and reservoir hosts. This is because adaptation to one host might alter interactions with a second host, and in some cases move a pathogen population away from its optimal virulence. In this study, we demonstrate the impact that short‐term pathogen adaptation to one plant host can have on growth in another plant species. Serial passage of *Pst* PT23 on either tomato (its native host) or *Arabidopsis* (an alternative host) resulted in different within‐host growth phenotypes, based on bacterial densities over time, when re‐inoculated onto either the passage host or the alternative host. This phenotypic difference was not simply the result of local adaptation to the passage host, whereby bacterial densities were higher in the original host than nonhosts, but rather was consistent across both plant species. This result indicates that selection within *Arabidopsis* lead to increased pathogen growth within both *Arabidopsis* and tomato relative to selection within tomato plants. This effect was not observed when bacterial lines were tested in tomato after the first passage only, suggesting it is not simply an effect of growth in a particular plant environment (e.g. a plastic response to one host). Moreover, neither of the plant‐selected lines showed increased growth rates relative to one another when grown in liquid media, implying a more complex adaptive outcome than metabolic processes alone. The results from our genomic screens also support this interpretation, with a number of mutations identified in plasmid conjugation transfer proteins and putative mobilization proteins, but none that were consistent across experimental treatments.

There are a number of reasons why *Arabidopsis* hosts may provide a different selective environment to tomato plants, including the tissue structure, immune system and microbiome (although note that these plants were grown from surface sterilized seeds in growth chambers, so this is unlikely to be a strong selective force within the current experiment). One example of a disparity in immune recognition is the flagellin receptor, a core component of plant and animal immune systems, which is known to differ between *Arabidopsis* and tomato (Robatzek *et al*. 2007) and could lead to selection for different epitopes. The production of hormone mimics in *Pst* is critical to subversion of the host immune response, allowing entry into the plant (Melotto *et al*. 2006), but this is again likely to differ quantitatively among host‐*Pst* interactions. Our results show that pathogen growth differs across the two plant species, with higher overall densities observed in tomato relative to *Arabidopsis*. This is not surprising given the differences in plant leaf morphologies, defences and co‐evolutionary history with this pathogen and is in line with previous evidence (e.g. Mittal & Davis 1995). However, whether these ecological differences in bacterial growth underpinned the observed evolutionary change (whereby 20 days of selection in *Arabidopsis* was enough to alter the growth rate of the bacteria in both *Arabidopsis* and tomato) remains to be determined. While all bacterial populations drastically reduced in size by the end of the assay experiment (i.e. by 192 h), we suspect that this is not the direct result of a successful immune response from either plant species. Watering of assay plants was stopped following inoculation to prevent transmission between experimental plants. As such, the reduction likely reflects tissue death of the plant or overexploitation from the bacterial populations, possibly as a result of the longer assay times than original transfer times. The effect of adaptation to one host on growth in alternative hosts has been previously demonstrated for the two‐spotted spider mite, *Tetranychus urticae* (Agrawal 2000; Magalhaes *et al*. 2007), and for experimentally evolved plant viruses (reviewed in Elena (2016). However, these effects do not appear to be universal, as the passage host of *cucumber mosaic virus* (CMV) did not seem to affect its fitness when grown on cucumber, bean and tomato, even after experimental evolution within the hosts of origin (Sacristán *et al*. 2005). More generally, the influence of host heterogeneity on pathogen evolution is a subject for which there is much more theoretical work than empirical investigation (Betts *et al*. 2016). This is unfortunate given the pressing need to better understand the role of host utilization, switching and sharing in shaping the emergence and spread of pathogens.

Despite the observed phenotypic divergence among tomato and *Arabidopsis*‐evolved lines, we observed no clear signatures of genomic change that could be explained by plant environment, nor evidence for parallel evolution among lines. This discrepancy between genotypic and phenotypic change over the course of experimental pathogen adaptation is in line with previous reports. Experimental evolution of tobacco etch potyvirus (TEV) uncovered a strong phenotypic signature of local adaptation to hosts, but only weak evidence for parallel evolution across lines, suggesting multiple mechanisms of host‐specific adaptation (Bedhomme *et al*. 2012). In contrast, serial passage of *Ralstonia* on distant (or reservoir) hosts did identify the repeated mutation of a single gene, despite very few genomic modifications overall (Guidot *et al*. 2014).

Surprisingly, we saw little effect of experimental evolution in vitro on subsequent growth within the host. This suggests that the previous adaptations to the host environment were not lost under relaxed selection (i.e. in the absence of a host immune system), which is itself suggestive of few if any costs associated with virulence. It is important to note, however, that the in vitro environment was both resource‐rich and lacking any among‐species/strain competition, and it is unclear whether a harsher in vitro environment would lead to loss of plant‐specific adaptations. However, an intriguing possibility is that the *P. syringae* species complex has evolved in a way that is robust to short‐term selection within nonhost environments that leads to loss of fitness within plants. We might predict this from the generalist lifestyle observed for this bacterium, which has been recovered from environmental samples ranging from agricultural soils to rain, snow, alpine streams and lakes (Morris *et al*. 2007, 2008). In contrast to the phenotypic results, the in vitro and *in planta* environments did seem to differ in the rate of molecular evolution of bacterial populations. Overall, the number of mutations we observed were lower for the in vitro populations than the *in planta* populations, despite similar population ‘bottlenecking’ at each transfer. Although the overall number of SNPs in our data set was higher than expected for such a short evolution experiment, suggesting the presence of false positives in the data set which are notoriously difficult to avoid without a ‘gold standard’ reference genome, all experimental lines were treated in the same manner from collection of isolates, through to library preparation and sequencing. Therefore, there would be no reason to expect a disproportionate number of false positives in the plant lines relative to the in vitro lines. Furthermore, previous evidence from *P. syringae* pv. *phaseolicola* uncovered rapid genomic change after just one passage through the plant environment (Lovell *et al*. 2011).

Interestingly, we found no effect of co‐inoculation with high titres of phages on bacterial adaptation. This was surprising, as phages have repeatedly been shown to be a key selective force on bacterial populations both in vitro and in the natural environment (Buckling & Rainey 2002; Rodriguez‐Valera *et al*. 2009). Direct trade‐offs between phage resistance and bacterial growth within the host can be due to changes in surface receptors, as well as indirect effects such as a reduction in bacterial population size and therefore pool of adaptive mutations available for selection (Gandon & Michalakis 2002; Filippov *et al*. 2011). Notably, the phage stock used to make the inoculum was still viable at the end of the experiment and the presence of phages (areas of obvious lysis on agar plates) was observed in some samples throughout the experiment. Despite the clear potential for phage‐mediated selection, however, we did not observe any impact of phage presence on the bacterial densities at the time of passaging (5 days after inoculation; Supplemental materials, Fig. S1, Supporting information) at any of the passages. Although we do not know what effect phages may have had during the initial pathogen colonization and growth phase (and preliminary evidence suggests this phage has its largest impact on bacterial growth at 24 h post‐inoculation), the lack of an observed difference in phenotype among the treatments suggests that phages had little impact on bacterial populations within this experiment. That being said, we did observe a slight decrease in the number of SNPs found in phage present vs. phage absent lines across selection environments, which is suggestive of bottleneck occurring during the initial colonization in phage present lines. Importantly, the streak assays we used to detect bacterial resistance to phage at the end of the experiment found no evidence for the evolution of resistance during passaging. Of the 12 colonies screened per line from the final transfer, only one line showed any resistance at all (and here, 83% of isolates were still sensitive). This was despite repeated co‐inoculation with phages, suggesting that any ecological effect phages imposed was not coupled by an evolutionary response by the pathogen. We later discovered this was in part due to a dramatic change in colony morphology that lead us to exclude the very rare colonies exhibiting the ‘smooth’ phenotype that can be indicative of phage resistance when preparing each inocula for passaging. Importantly, Sanger sequencing of our colonies found no false positives in our identification of *P. syringae* colonies, but some false negatives, that is five of 10 colonies with uncharacteristic morphologies that we classified as nonpseudomonas (and therefore excluded from passaging) were in fact *P. syringae*, with the remainder being *Enterobacteria*,* Curtobacteria* and *Sphingomonas*. As such, any experimental effect of phage on adaptation within this experiment would be strictly ecological (e.g. decreased density of the sensitive bacterial population) rather than evolutionary (e.g. trade‐offs associated with resistance). We previously demonstrated that the evolution of phage resistance can be costly (Meaden *et al*. 2015), especially in the tomato plant environment. As such, it is possible that sensitive bacteria, surviving the initial co‐inoculation, outcompete resistant bacteria once the phage population has become extinct. Further insight into this unexpected phenomenon will require further study.

In summary, our results emphasize the impact that within‐host evolution in one plant species can have on pathogen growth within another. As the pathogen strain used in this experiment had not previously been whole genome sequenced, we also present a novel genome for the *Pst* strain PT23 that we hope will allow further comparative genomic studies. Using this new genomic data, we were able to measure mutational change over the course of 20 days of selection either *in planta *or in vitro. We observed numerous mutations, but were unable to identify changes that were consistently associated with the observed phenotypic changes across lines. Together, our results highlight the utility of the experimental evolution and resequence approach for understanding pathogen adaptation to novel hosts, especially for pathogens with broad host range potential. The power of comparative genomics to uncover genetic change associated with host‐specific adaptation has been known for some time (e.g. Tyler *et al*. 2006; Baltrus *et al*. 2011) but whether such genotype–phenotype associations are observed over more rapid experimental evolutionary timescales remained unclear. Our results therefore lend caution to the idea that sequencing results can offer insight to pathogen adaptation over short evolutionary timescales, even when rapid phenotypic change is observed. Overall, while identifying genes implicated in virulence is crucial, a broader understanding of the evolution of virulence in reservoir hosts is required if accurate prediction of landscape‐level pathogen‐mediated plant mortality is to be successful.

S.M. and B.K. designed the experiment. S.M. led all empirical examination and bioinformatics analyses, and both S.M. and B.K. wrote the study.

## Data accessibility

Data are available from the Dryad Digital Repository: https://doi.org/10.5061/dryad.v444t, and the PT23 genome assembly is available under BioProject ID PRJNA357646 on the NCBI database.

## Supporting information


**Fig. S1** Bacterial densities for plant lineages at each transfer during the passage experiment. Co‐inoculation with phages had no significant overall effect on bacterial densities at 5 days post‐infection.
**Fig. S2** Bacterial densities for plant lineages at each transfer during the passage experiment. The plant environment did influence bacterial densities at 5 days post‐infection over the course of the experiment.
