# Supplementary material for: Adaptation of the pathogen, Pseudomonas syringae, during experimental evolution on a native vs. alternative host plant
Source: Mol Ecol. 2017 Mar 13;26(7):1790–801. doi: 10.1111/mec.14060 (PMC6849854; doi:10.1111/mec.14060)
Supplement: Supplementary file 1 — Fig. S1 Bacterial densities for plant lineages at each transfer during the passage experiment. Co‐inoculation with phages had no significant overall effect on bacterial densities at 5 days post‐infection. Fig. S2 Bacterial densities for plant lineages at each transfer during the passage experiment. The plant environment did influence bacterial densities at 5 days post‐infection over the course of the experiment. [file MEC-26-1790-s001.docx]

**Adaptation of the pathogen, *Pseudomonas syringae* during experimental evolution on a native versus alternative host plant**

Sean Meaden^1,2^ * and Britt Koskella^2^

^1^ University of Exeter, Penryn campus, Penryn, Cornwall, TR11 4EH, UK

^2^ University of California, Berkeley, Department of Integrative Biology, Berkeley, CA, 94720, USA

* Corresponding author: S.Meaden@exeter.ac.uk

Supplementary Information

*Fig S1. Bacterial densities for plant lineages at each transfer during the passage experiment. Co-inoculation with phages had no significant overall effect on bacterial densities at 5 days post-infection. Data represent samples from both host plant species pooled (n = 36, error bars = 1SE). Repeated measures analysis found a main effect of time (*F_3,13_= 8.106, P= 0.003*), but no main effect of phage (*F_1,15_= 1.652, P= 0.218).

*Fig S2. Bacterial densities for plant lineages at each transfer during the passage experiment. The plant environment did influence bacterial densities at 5 days post-infection over the course of the experiment. Data represent samples from both phage and no phage samples pooled (n = 36, error bars = 1SE). Repeated measures analysis found a main effect of time (*F_3,13_= 8.106, P= 0.003*), and of plant host environment (*F_1,15_= 8.915, P= 0.009).
